# Sympathetic nerve innervation and metabolism in ischemic myocardium in response to remote ischemic perconditioning

**DOI:** 10.1007/s00395-022-00946-3

**Published:** 2022-08-25

**Authors:** Attila Kiss, Ping Wu, Michaela Schlederer, Patrick M. Pilz, Petra Lujza Szabo, Jingle Li, Lukas Weber, Chrysoula Vraka, Verena Pichler, Markus Mitterhauser, Xiaoli Zhang, Karin Zins, Dietmar Abraham, Sijin Li, Bruno K. Podesser, Marcus Hacker, Xiang Li

**Affiliations:** 1 Ludwig Boltzmann Institute for Cardiovascular Research at the Center for Biomedical Research, Medical University of Vienna, Vienna, Austria; 2 Division of Nuclear Medicine, Department of Biomedical Imaging and Image-Guided Therapy, Medical University of Vienna, Währinger Gürtel 18-20, Floor 3L, 1090 Vienna, Austria; 3 Department of Nuclear Medicine, Collaborative Innovation Center for Molecular Imaging of Precision Medicine, First Hospital of Shanxi Medical University, Taiyuan, China; 4 Department of Experimental and Translational Pathology, Institute of Pathology, Medical University of Vienna, Vienna, Austria; 5 Division of Pharmaceutical Chemistry, Department of Pharmaceutical Sciences, University of Vienna, Vienna, Austria; 6 Ludwig Boltzmann Institute for Applied Diagnostics, Vienna, Austria; 7 Laboratory for Molecular Imaging, Department of Nuclear Medicine, Beijing Anzhen Hospital, Capital Medical University, Beijing, China; 8 Center for Anatomy and Cell Biology, Medical University of Vienna, Vienna, Austria

**Keywords:** Myocardial ischemia/reperfusion, Remote ischemic perconditioning, Cardiac sympathetic nerve, Cardiac metabolism

## Abstract

Sympathetic nerve denervation after myocardial infarction (MI) predicts risk of sudden cardiac death. Therefore, therapeutic approaches limit infarct size, improving adverse remodeling and restores sympathetic innervation have a great clinical potential. Remote ischemic perconditioning (RIPerc) could markedly attenuate MI-reperfusion (MIR) injury. In this study, we aimed to assess its effects on cardiac sympathetic innervation and metabolism. Transient myocardial ischemia is induced by ligature of the left anterior descending coronary artery (LAD) in male Sprague–Dawley rats, and in vivo cardiac 2-[^18^F]FDG and [^11^C]*m*HED PET scans were performed at 14–15 days after ischemia. RIPerc was induced by three cycles of 5-min-long unilateral hind limb ischemia and intermittent 5 min of reperfusion during LAD occlusion period. The PET quantitative parameters were quantified in parametric polar maps. This standardized format facilitates the regional radioactive quantification in deficit regions to remote areas. The ex vivo radionuclide distribution was additionally identified using autoradiography. Myocardial neuron density (tyrosine hydroxylase positive staining) and chondroitin sulfate proteoglycans (CSPG, inhibiting neuron regeneration) expression were assessed by immunohistochemistry. There was no significant difference in the mean hypometabolism 2-[^18^F]FDG uptake ratio (44.6 ± 4.8% vs. 45.4 ± 4.4%) between MIR rats and MIR + RIPerc rats (*P* > 0.05). However, the mean [^11^C]*m*HED nervous activity of denervated myocardium was significantly elevated in MIR + RIPerc rats compared to the MIR rats (35.9 ± 7.1% vs. 28.9 ± 2.3%, *P* < 0.05), coupled with reduced denervated myocardium area (19.5 ± 5.3% vs. 27.8 ± 6.6%, *P* < 0.05), which were associated with preserved left-ventricular systolic function, a less reduction in neuron density, and a significant reduction in CSPG and CD68 expression in the myocardium. RIPerc presented a positive effect on cardiac sympathetic-nerve innervation following ischemia, but showed no significant effect on myocardial metabolism.

## Introduction

Myocardial infarction (MI) remains the most common cause of heart failure (HF) and it is still a leading cause of mortality and morbidity worldwide. Besides inducing cardiomyocytes death, transmural MI damages sympathetic-nerve (SN) fibers running through the infarct zone and results in regional sympathetic denervation [4, 12]. Furthermore, the SN is more vulnerable to ischemic insult than cardiomyocytes and SN denervation highly predictive of ventricular arrhythmias and sudden cardiac [15, 21]. Despite the robust development of early revascularization and pharmacological therapy, cardiac arrhythmias and contractile dysfunction represent common consequences of remodeling and most serious complications among patients with MI [38]. Therefore, novel therapeutic strategies that effectively mitigate or reverse adverse post-infarcted cardiac remodeling and risk of sudden cardiac death are unmet need to develop. One of the most investigated cardioprotective strategy is called ischemic conditioning. In addition, remote ischemic conditioning (RIC) by applying transient, repetitive ischemia–reperfusion (IR) interventions at distant tissues or organs has emerged as a novel cardiac protective strategy to achieve protection against acute MI injury in the heart through a novel neuro and humoral pathway [26]. In general, neuronal pathway is the neuronal reflex as peripheral stimuli are processed in the brain and efferent sympathetic and parasympathetic fibers innervate the myocardium; the humoral hypothesis describes the capability of various mediators which are released during RIC procedure in the periphery and transferred by bloodstream, to protect the heart against IR injury [22, 33]. The activation of peripheral sensory nerves and then the sensory afferent transduction by RIC projected into autonomic centers of the central nervous system where the changeover to efferent vagal nerve is proceeded, and consequently, the activation of vagal nerve and downstream signaling pathways refers to cardioprotection [22]. In addition, a recent pioneering study by Kleinbongard et al*. *[32] further elucidated the potential importance of neuronal pathway in the cardioprotection of RIPerc in a clinically relevant pig model of myocardial infarction.

There are numbers of proof of concept clinical studies which demonstrated that remote ischemic postconditioning (RIPost) alone or combination with remote ischemic perconditioning (RIPerc) markedly improved adverse remodeling and infarct size among patients with ST-segment elevation myocardial infarction (STEMI) [11, 19, 46]. In contrast, large clinical studies failed to show the cardioprotective effect of remote ischemic preconditioning (RIPrec) in patients subjected to elective cardiac surgery [23, 37]. Furthermore, other clinical study also failed to demonstrate the cardioprotective benefit of RIC in STEMI patients [24].

In general, nerve injury, e.g., acute MI causes the ‘activation’ of peripheral glia, and the Schwann cells (SCs), which repair-specific functions affect the whole microenvironment, may provide tissue-specific support in injured organs [5, 10]. In addition, Neureguin-1 (NRG1) and nerve growth factor-mediated signaling may also be crucial for the proliferation, survival, motility, and differentiation of SCs following a myocardial injury such as MI [9]. In line with previous reports, our group proved the positive value of RIPerc in the rat model of myocardial IR injury, associating with an increase of cardiac NRG1 expression [41]. Furthermore, previous studies also demonstrated that the delay of cardiac sympathetic reinnervation as well as the expansion of denervated infarcted myocardium significantly increase the risk of sudden cardiac death after MI [18, 25]. Collectively, cardiac sympathetic-nerve reinnervation may be an encouraging therapeutic benefits. However, there is limited approach to improve cardiac denervation after MI.

Regional quantification of cardiac SN function, in conjunction with an assessment of regional viability, is essential for investigating ischemia injury. Cardiac positron emission tomography (PET) utilizing 2-deoxy-2-[^18^F]fluoro-d-glucose (2-[^18^F]FDG) showed superior sensitivity for myocardial metabolism assessment. Of importance, PET with the ^11^C-labeled norepinephrine analog [^11^C]*meta*-Hydroxyephedrine ([^11^C]*m*HED) enables non-invasive idenfication of local cardiac SN injury in patients with HF and ischemic cardiomyopathy [1, 36]. Altogether, these reports indicate that the delay of cardiac SN innervation in infarcted and peri-infarcted tissue may be considered an essential component and surrogate marker of post-MI adverse left-ventricular (LV) remodeling and sudden cardiac death. Therefore, we assessed the effects of RIPerc on cardiac innervation and metabolism utilizing PET imaging plus immunochemistry verification, aiming to provide key evidence in developing novel pharmacological or non-pharmacological approaches for boosting early innervation, and further in reduction of cardiac mortality in patients with MI.

## Methods

### Animals

Male Sprague–Dawley rats (10–12 weeks old, 250–300 g, Department for Laboratory Animal Science and Genetics, Himberg, Austria) were used. The experimental protocol was approved by the Regional Ethics Committee for Laboratory Animal Experiments at the Medical University of Vienna and the Austrian Ministry of Science Research and Economy (BMWFW-66.009/xxx-WF/V/3b/2016), and conforms with the Guide for the Care and Use of Laboratory Animals, published by the US National Institutes of Health (NIH Publication No. 85–23, revised 1996).

### Myocardial ischemia and reperfusion (MIR) injury in vivo

**Fig. 1:**
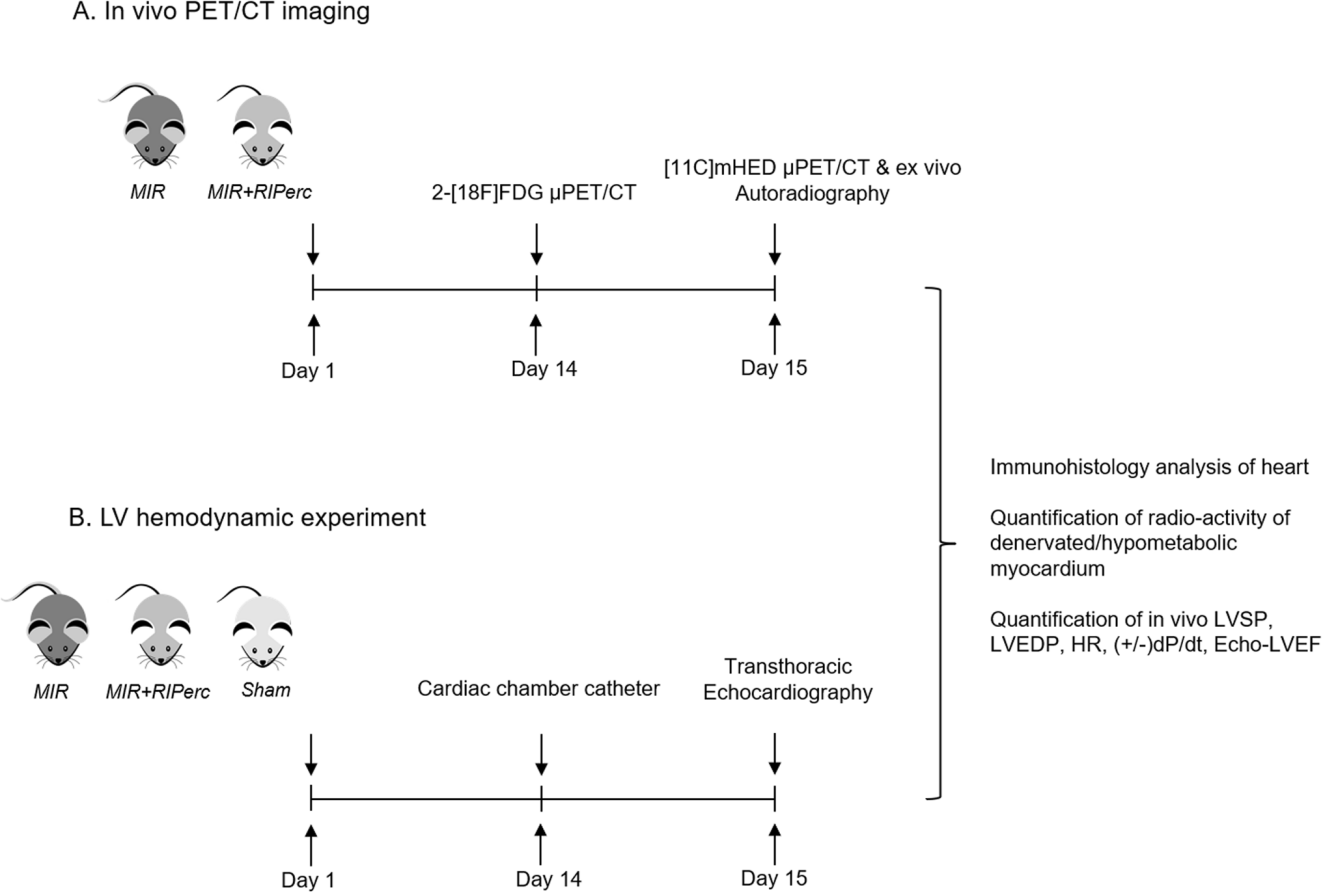
Working flow of study. MIR: myocardial ischemia and reperfusion**.** *LVSP* left-ventricular systolic pressure, *LVEDP* left-ventricular end-diastolic pressure, *(±)dP/dt* peak velocities of pressure change, *HR* heart rate, *LVEF* left-ventricular ejection fraction

Briefly, rats were anesthetized by intraperitoneal injection of a mixture of Xylazine (4 mg/kg; Bayer, Germany) and Ketamine (100 mg/kg; Dr E. Gräub AG, Switzerland), intubated (14-gauge tube), and ventilated (9 ml/kg body weight, 75–85 stroke/min). Rectal temperature was measured and maintained at 37.5–38.5 °C by a heated operating table. The heart was exposed via a left thoracotomy and a ligature was placed around the left anterior descending coronary artery (LAD) 2–3 mm away from the origin for 30 min, followed by 14–15 days of reperfusion as described previously in rats [41]. Myocardial ischemia was associated with the pallor of the myocardial area at risk and ST elevation on ECG signal. Sham rats served as controls were those completed thoracotomy but without LAD occlusion. Reperfusion was initiated following the 30 min of LAD occlusion by removal of the snare. RIPerc was induced by three cycles of 5 min of IR on hindlimb performed during myocardial ischemia as described previously [47]. Analgesia was initiated by intraperitoneal injection of Piritramide (0.1 ml/kg body weight) preoperatively and piritramide was applied in drinking water as a postoperative analgesic regimen (2 ampules of Piritramide with 30 ml of Glucose 5% in 250 ml water). Experimental protocol is depicted in Fig. 1.

### Small animal PET/CT imaging

Fourteen days after the reperfusion, µPET scans were performed. A dedicated small animal µPET/µCT system (Inveon, Siemens Medical Solutions Inc., Erlangen, Germany) was employed. All animals were maintained under anesthesia throughout the imaging acquisition. 10 min insulin-stimulated 2-[^18^F]FDG PET/CT (30–40 Mbq) acquisition were conducted 60 min after tracer injection, and the 10 min static [^11^C]*m*HED PET/CT (40–50 Mbq, 30 min after tracer injection) was performed on a subsequent day. All data were acquired in three-dimensional (3D) and sorted into a 3D sonogram of 16 frames per cardiac cycle. The sinogram was reconstructed into a 128 × 128 × 95 voxel image by 2D-filtered back-projection image reconstruction using a ramp filter with the Nyquist limit (0.5 cycles/voxel) as the cut-off frequency. The voxel size equaled 0.43 × 0.43 × 0.80 mm. Data were normalized and corrected for randoms, dead time, and decay. An attenuation correction transmission scan was additionally performed before the µPET scan.

### Imaging analysis

Late uptake defined the LV with bottle-brush sampling. Late myocardial uptake was averaged from 4 frames of data for each imaging 15–30 min after [^11^C]*m*HED injection and 45–60 min after 2-[^18^F]FDG injection. Using the volumetric sampling of the cardiac PET/CT scans, we defined myocardial radioactivity LV sectors and semi-automatically constructed radioactivity into cardiac metabolism and nerve polar maps. Each polar map was normalized to a region of interest (ROI) in the remote myocardium (inferior wall) at the ventricular level. The metabolism deficit or the denervation area in the myocardium was defined as 2-[^18^F]FDG or [^11^C]*m*HED uptake ≤ 60% of maximum activity. The uptake ratio in the defected area was defined as SUVR = mean of standardized uptake value (SUV_mean_)/remote control myocardium uptake (SUV_mean_). All µPET images were analyzed using the MunichHeart/NM software package.

### Autoradiography

The heart was excised immediately for radioassay after assessment of LV hemodynamics. Ex vivo accumulation of myocardial [^11^C]*m*HED was assessed using 20 μm sections of sample tissues with a digital autoradiography system (Cyclone^®^ Plus Storage Phosphor System; PerkinElmer). Myocardial defect area was determined by a threshold of 60% of maximum myocardial activity in midventricular slices; the radioactivity values of the placed ROIs were drawn on short-axis slices and then were expressed as background-corrected photostimulated luminescence units per area. Measured values were normalized to account for differences in the amount of tracer injected. Then, the uptake activity of the defect area was calculated as percentages in relation to the remote area on the short-axis slice.

### Assessment of LV hemodynamics

LV hemodynamic function was performed in vivo invasively as described previously [48]. In brief, rats were anesthetized by intraperitoneal injection of a mixture of Xylazin (4 mg/kg; Bayer, Germany) and Ketamin (100 mg/kg; Dr E. Gräub AG, Switzerland), intubated, and ventilated. The chest was opened and a microtip catheter (SPR-409, 2F, Millar Instruments, Houston, USA) was gently inserted into the LV chamber. Hemodynamic parameters such as LV systolic pressure (LVSP), LV end-diastolic pressure (LVEDP), and heart rate (HR) were continuously recorded on Labchart (v7.3.2, Powerlab System (8/30, both AD Instruments, Spechbach, Germany) connected to a Bridge Amplifier (Bridge Amp, ADInstruments, GmbH, Spechbach, Germany) and a Transducer Control unit (Model TC-510, Millar Instruments Inc., Houston, Texas, USA). In addition, transthoracic echocardiography was performed as described previously [41]. Briefly, rats were anesthetized (isoflurane 2–3%) and echocardiography was performed prior to sacrifice the rats using Vivid7 system (GE Healthcare, USA) equipped with an 11.5 MHz 10S sector transducer. Left-ventricular ejection fraction (LVEF) was evaluated.

### Histology and immunochemistry

Tyrosine hydroxylase (TH) positive stain was performed to access the density of the neurons in myocardium. In addition, the reduction of cardiac neurons after MIR was shown to link a transient overexpression of chondroitin sulfate proteoglycans (CSPG, inhibiting neuron regeneration) in the animal hearts [18]. Therefore, we assessed immunochemistry staining for CSPG (within the infarcted area). Immunohistochemistry was also performed to evaluate the influx of CD68 + macrophages. Formalin-fixed paraffin-embedded LV cardiac tissue sections were hematoxylin and eosin (HE) stained. Streptavidin–biotin immunostaining for CD68 (1:100; mouse monoclonal, ED1, ab31630, Abcam, Cambridge, MA, USA), TH (1:100; rabbit monoclonal, EP1533Y, Abcam, Cambridge, MA, USA), and CSPG (1:500; mouse monoclonal, clone Cat-301, MAB5284, Merck, Darmstadt, Germany) were used as described previously [48]. Briefly, primary antibodies were detected with appropriate biotinylated secondary antibody (Vector Laboratories, Burlingame, CA) and horseradish peroxidase (HRP) conjugated streptavidin (Dako, Glostrup, Denmark), developed with 3,3′-diaminobenzidine (DAB) (Vector Laboratories), counterstained with hematoxylin, dehydrated, and mounted in DPX (Merck, Darmstadt, Germany). TH was developed with 3-Amino-9-ethylcarbazole (AEC) under visual control, counterstained with hematoxylin, and mounted with Aquatex (Merck, Darmstadt, Germany). Digitalized images were generated with a Nikon Eclipse 80i (Tokyo, Japan) microscope. Multiple images were obtained from each slide using × 40 objective and slides of each group were randomly selected for quantification of CD68 + macrophages and CSPG-positive areas. CD68 + macrophages were counted and results were expressed as cells per section. Quantification of CSPG-positive area in each field of view was performed using ImageJ software to measure the ratio between the positive area and the total image [31].

### Statistics

Parametric data of the three groups are presented as mean ± standard deviation (SD). Statistical analyses were performed with SPSS 24 and GraphPad Prism Software (version 7.03; GraphPad Software Inc., San Diego, CA, USA). Differences were evaluated by one-way ANOVA followed by Bonferroni post hoc test or Students’ paired *t* test when appropriate. *P* values of < 0.05 were considered statistically significant.

## Results

### PET imaging

**Fig. 2:**
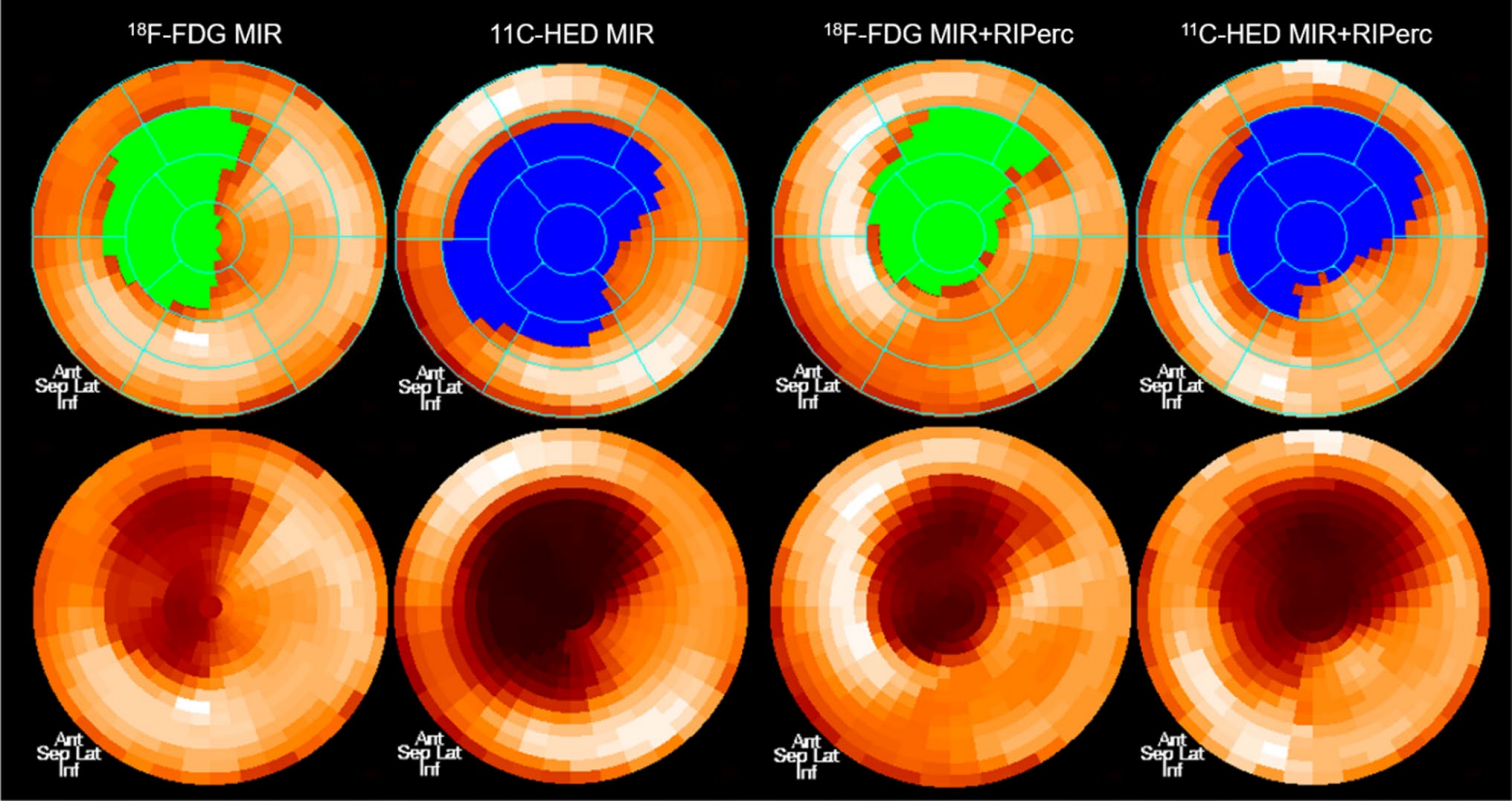
Polar maps of 2-[^18^F]FDG ( myocardial viability) and [^11^C]*m*HED (sympathetic innervation) in representative animal with MIR and MIR + RIPerc. The denervation (downregulated [^11^C]*m*HED uptake) volume (%) is larger than the infarct volume (%) (defected 2-[^18^F]FDG uptake) = 51.2(MIR) vs. 28.2(MIR); 37.3(MIR + RIPerc) vs. 30.9(MIR + RIPerc), respectively. Correspondingly, these two animals presented myocardial [^11^C]*m*HEDuptake ratio SUVR_mean_(MIR + RIPerc) = 35.6 vs. SUVR_mean_(MIR) = 26.0 and [^18^F]FDG uptake ratio SUVR_mean_(MIR + RIPerc) = 36.4 vs. SUVR_mean_(MIR) = 44.5

Quantitative cardiac polar map of viability (2-[^18^F]FDG), and sympathetic innervation [^11^C]*m*HED PET images from 2 representative subjects (MIR vs MIR + RIPerc) are presented in Fig. 2. The metabolism-deficit 2-[^18^F]FDG area/remote area uptake ratio in the two groups (NO.: MIR = 9 vs. MIR + RIPerc = 9) showed no difference (MIR vs. MIR + RIPerc: SUVR_mean_ = 44.6 ± 4.8 vs. 45.4 ± 4.4, *P* > 0.05) and there was no difference of hypometabolism area volume/global ventricular volume between MIR group and MIR + RIPerc groups (14.7 ± 4.7% vs.15.3 ± 2.5%, *P* > 0.05). However, the relative [^11^C]*m*HED uptake ratio (%) in denervated myocardium to remote area was significantly elevated in the MIR + RIPerc group in comparison to the MIR group (SUVR_mean_ = 35.9 ± 7.1 vs. 28.9 ± 2.3, *P* < 0.05), and the denervated area/global ventricular area was significantly decreased in MIR + RIPerc animals vs MIR group (19.5 ± 5.3% vs. 27.8 ± 6.6%, *P* < 0.05).

### Autoradiography

**Fig. 3:**
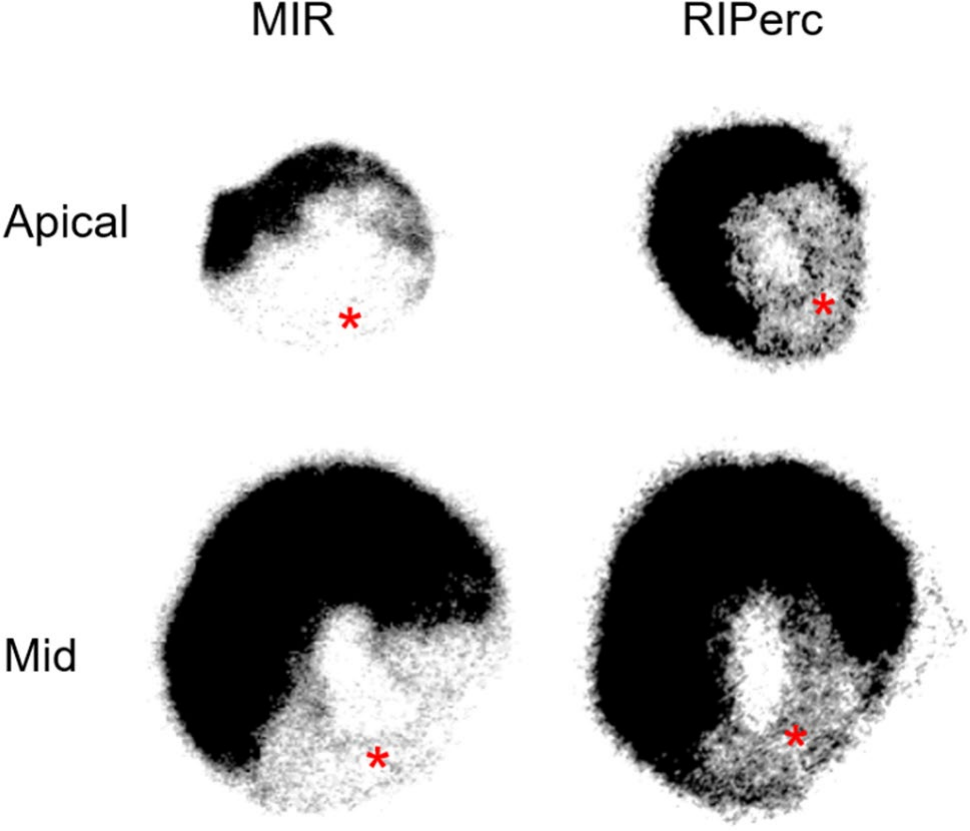
Autoradiography of [^11^C]*m*HED in representative rats with MIR and MIR + RIPerc. Relatively increased sympathetic innervation was found in the defect area (*) of rats with MIR + RIPerc

Ex vivo [^11^C]*m*HED uptake ratios to the remote control region were 0.30 ± 0.14 (MIR) and 0.39 ± 0.17 (MIR + RIPerc), respectively (*P* < 0.01). Representative autoradiography is shown in Fig. 3.

### Left-ventricular hemodynamic function

As displayed in Table 1, decreased LVSP, increased LVEDP, and reduced peak velocities of pressure change during isovolumic contraction (+ d*P*/d*t*) and isovolumic relaxation (− d*P*/d*t*) in rats with MIR were observed in comparison to the sham group (*P* < 0.01, respectively). These parameters were improved by RIPerc, markedly in LVSP and + dP/dt. No differences between the groups were observed in HR. In addition, LVEF significantly declined in MIR in comparison to Sham-OP (83 ± 4% vs 66 ± 4%; *n* = 5 and 6, respectively, *P* < 0.001). Rats with RIPerc showed a preserved LVEF (72 ± 2% vs 66 ± 4%; *n* = 5 and *n* = 6 respectively, *P* < 0.05 vs MIR), suggesting the benefit of RIPerc on adverse cardiac dysfunction.

**Table 1:** Left-ventricular hemodynamic parameters

Parameter/group	Sham-OP	MIR	MIR + RIPerc
n	4	5	6
LVSP (mm Hg)	117 ± 5	83 ± 5***	95 ± 8^#^
LVEDP (mm Hg)	2.46 ± 0.50	5.70 ± 1.49***	4.18 ± 0.63
+ d*P*/d*t* (mm Hg/s)	7074 ± 564	4255 ± 380***	5139 ± 588^#^
−d*P*/d*t* (mm Hg/s)	− 5879 ± 478	− 3125 ± 873**	− 3827 ± 669
HR (bpm)	258 ± 28	257 ± 40	244 ± 28

*LVSP* left-ventricular systolic pressure, *LVEDP* left-ventricular end-diastolic pressure, *( ±)dP/dt* peak velocities of pressure change, *HR* heart rate

Values are mean ± SD; **P < 0.01 and ***P < 0.001 Sham vs MIR, and ^#^ *P* < 0.05 MIR vs MIR + RIPerc

### Immunochemistry

**Fig. 4:**
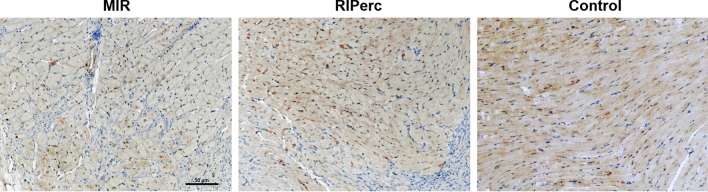
Immunochemistry of tyrosine hydroxylase (TH) positive neurons in the myocardium. Representative images show TH staining in the anterolateral area of the defect area (at scar region) in the left-ventricular wall in MIR and RIPerc rats. TH-positive signal is extensively reduced in MIR rats after myocardial infarction compared to control area, and less reduction in RIPerc rats

**Fig. 5:**
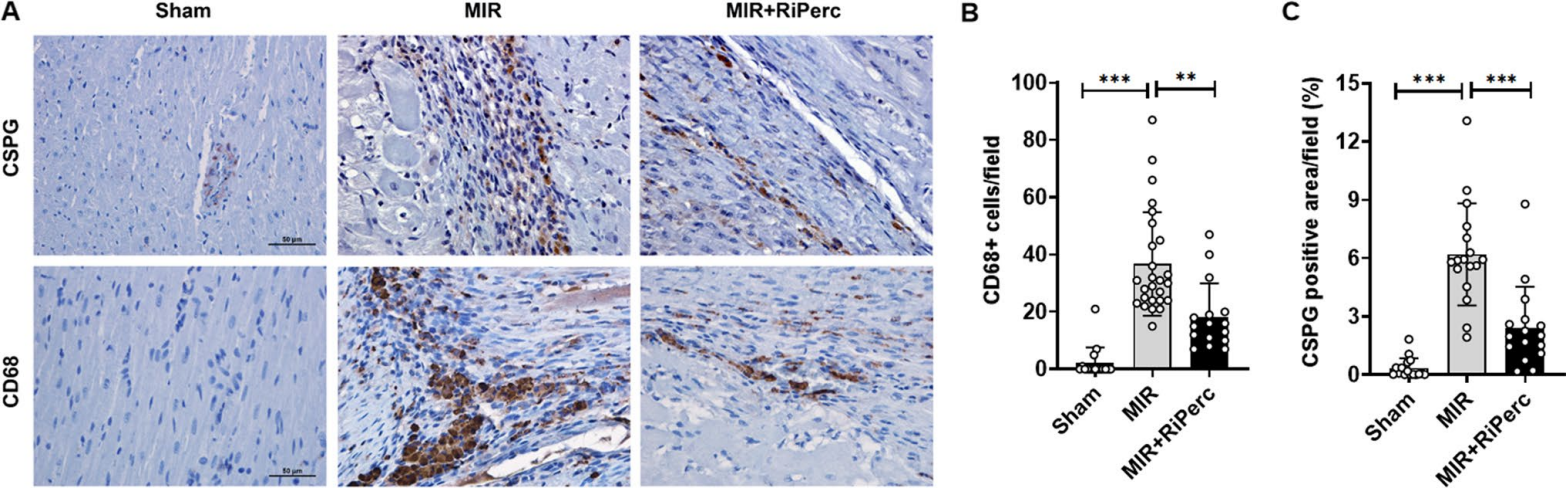
Effect of remote ischemic perconditioning on influx of CD68 + cells, and CSPG-positive area expression**.** **A** Photomicrographs of immunohistochemical staining of CD68 and CSPG in myocardial sections of hearts from Sham, MIR and MIR + RiPerc 2 weeks after MIR. Panels are from border zone of MI. Histogram of **B** CD68 and **C** CSPG immunoreactive cells and area, respectively. Data are mean ± SD of Sham (*n* = 4), MIR (*n* = 7), and MIR + RIPerc (*n* = 5)

There is a more prominent reduction in TH-positive neurons in MIR rats in comparison to MIR + RIPerc rats (Fig. 4). In comparison to Sham rats, the expression of CSPG was markedly increased in MIR rats (Fig. 5), particularly in the infarcted area and border zone. In contrast, in MIR + RIPerc rats, the CSPG expression was similar to the Sham group (Fig. 5). No CD68 + cells were detected in the Sham group (Fig. 5). In contrast, high numbers of CD68 + macrophages were present in the infarcted myocardial tissue of the MIR group, whereas MIR + RIPerc was associated with a reduced influx of CD68 + macrophages (Fig. 5).

## Discussion

Alterations of cardiac sympathetic-nerve innervation in cardiac diseases have been increasingly noticed. Nevertheless, a therapeutic approach to alleviate cardiac sympathetic dysfunction is limitedly available.

Cardiac sympathetic and parasympathetic innervation plays multiple roles in cardiac physiology and pathophysiology. There is large body of evidence that selective parasympathetic, e.g., vagus nerve stimulation during acute myocardial infarction protects the heart against myocardial IR injury and chronic stimulation of vagus nerve resulted in an improvement on post-MI remodeling and chronic heart failure, respectively [43, 51]. However, large clinical trials failed to show similar benefits in patients with congestive HF [3].

On the other hand, sympathetic hyperinnervation or denervation in the myocardium is connected to the progression of cardiac dysfunction [40, 42]. Sympathetic denervation typically occurs in the infarcted myocardium and is associated with sudden cardiac death [14, 17]. Impaired innervation was also demonstrated in non-infarcted myocardium in patients with ischemic and dilated cardiomyopathy [42]. Factors affecting SN integrity in infarcted and remote myocardium are largely unknown. Nevertheless, a recent pioneering study by Siebert et al. [44] demonstrated that CSPG expression within the infarcted myocardium delays the sympathetic innervation following MI. Therefore, novel non-invasive methods are urgently needed to visualize cardiac sympathetic (re)innervation in cardiovascular disease and find therapeutic approach to alleviate cardiac sympathetic dysfunction.

Multiple shreds of evidence demonstrated that a portion of dysinnervated but viable myocardium is essential to attenuate cardiac remodeling, improves cardiovascular function [16, 45]. In contrast, denervated but viable myocardium adjacent to infarcted myocardium may be particularly arrhythmogenic and prone to induce ventricular fibrillation and substantial risk for sudden cardiac death [49]. Thus, the non-invasive approach to identify nerve denervation in viable myocardium and scare area is crucial in patients with ischemic heart disease due to MI and may provide surrogate a prognostic markers for the risk for sudden cardiac death. Recent advantages in cardiac molecular imaging allow for the specific identification of synaptic biomarkers in the cardiac autonomic nervous system.

In the present study, we hypothesized that RIPerc alleviates cardiac sympathetic denervation following myocardial infarction. This was quantified using the state-of-the-art PET imaging in a rat model of myocardial ischemia and chronic reperfusion. There are numbers of study demonstrated the cardioprotective effects of RIC, e.g., preventing cell death of cardiomyocytes in acute MI. Moreover, Gho et al*.* demonstrated for the first time that the ganglion blocker hexamethonium abolishes cardioprotection established by RIC, suggesting the functional importance of neural (autonomic) pathways mediating this phenomenon [20]. Beside the infarct size limiting effect of RIC, there are only few studies investigated the impact of RIC on post-MI remodeling [41, 50] and no evidence whether RIC acts on SN activity and denervation in the subacute or chronic phase of MI. Therefore, we first assessed IR injury-mediated SN degradation and myocardial viability defection in a rat model of MIR. Interestingly, RIC presented cardiac protection against sympathetic denervation but has no protecting effect on viable myocardial stunning. These findings demonstrate that the SN might be more susceptible to ischemia injury than the cardiomyocytes. The remaining viable myocardium may preserve elevated SN fibers in RIPerc rats and substantially may preserve cardiac contractile function. In line with that, RIPerc group showed significantly higher LVSP and a tendency to improve diastolic dysfunction (LVEDP) compared to the MIR group as well as showing preserved LVEF, suggesting its benefit on cardiac pump function and adverse post-MI remodeling.

During acute myocardial ischemia, damage to cardiac sympathetic neurons results in the release of norepinephrine into the myocardial interstitial space [2]. In addition, other study demonstrated that after acute dissection of LAD the left ventrolateral cervical cardiac nerve stimulation resulted in increased shortening velocity of the posterial wall in dogs [29]. In the current study, we have not investigated whether cardiac SN reinnervation following myocardial IR injury is associated with the changes of regional contractile (dys)function. However, further studies are needed to clarify this important issue.

In general, the neuronal damage could be transient or irreversible, depending on the severity and duration of ischemia, and reversible SN dysfunction has been reported, but is slow in patients after MI [17, 33, 42].

Sympathovagal (im)balance is a critical component in the extent of myocardial damage and infarct size in acute MI. Indeed, previous studies demonstrated the critical importance of brain–heart and vago-splenic axis in acute cardioprotection by RIC [6, 35].

However, there is no evidence whether RIC affects myocardial denervation following MI. Chronic elevation of SN activity in the myocardium is a key component of the altered signaling pathways that accompany cardiac remodeling, increasing the risk for sudden cardiac death resulting from hypertension and acute MI [17, 40]. However, denervated myocardium and decrease in SN activity may play a pivotal role in impaired repair and regeneration following cardiac injury due to MI. In addition, recent studies [7, 18] suggest that early sympathetic-nerve denervation increases the risks for sudden cardiac death after MI. Thereby therapeutic manipulation which alleviate cardiac denervation or boost earlier SN reinnervation after MI may result in clinical benefit.

Furthermore, next to the myocardium, there is growing evidence that coronary circulation may undergo denervation after myocardial IR injury; hence, the coronary circulation is substantially affected by sympathetic-nerve activation. Accordingly, Heusch et al. [28] demonstrated that severe coronary artery stenosis significantly stimulates the sympathetic activation and subsequently aggravates myocardial ischemia in dogs. The underlying signaling mechanisms are mediated via alpha-adrenoceptor-mediated effects of sympathetic activation [27, 30]. In addition, Di Carli et al. [13] demonstrated that increases in coronary blood flow in response to sympathetic stimulation correlated with the regional norepinephrine content in the cardiac sympathetic-nerve terminals in transplanted human hearts. This provides a unique platform for studying the vasomotor responses to sympathetic-nerve stimulation in reinnervated heart. In our study, we have not assessed coronary/myocardial blood flow change in correlation to sympathetic-nerve reinnervation post-myocardial infarction with/without RIPerc. Therefore, further studies are warranted to clarify whether the sympathetic reinnervation and activation is associated with a subsequent changes of myocardial blood flow.

With injury or disease, an increase in extracellular CSPG expression is commonly observed close to lesion areas in the brain and spinal cord [44]. A more recent study showed that CSPG present in the cardiac scar after MI prevents sympathetic innervation by binding the neuronal protein tyrosine phosphatase receptor σ (PTPσ) and downstream signaling pathway activation increases the risk for sudden cardiac death [18]. Notably, we found that CSPG expression within the infarction area was markedly increased in rats with MIR, but it expression was substantially decreased in RIPerc animals. Since CSPG overexpression is linked to the increase risk of sudden cardiac death after MI [7], we hypothesize that the reduction of CSPG by RIPerc provides a beneficial effect of long-term survival in patients with MI. However, to translate this, important novel findings as well as benefit of RIPerc to clinic further rigorous and systematic preclinical studies are warranted [34].

Sympathetic denervation in patients with ischemia and reperfusion has been extensively recognized, but the potential effect of reversible SN dysfunction is less widely appreciated. We demonstrated for the first time that RIPerc markedly improved cardiac sympathetic-nerve denervation after MIR, and this was associated with a marked reduction of CSPG expression and inflammation.

### Limitations

Certain limitations of our study need to be acknowledged. Gated cardiac perfusion, scans were not performed to determine the cardiac contraction due to high kinetic positron energy and consequently extensive positron range for small animal images. Myocardial infarct size assessment by histology/immunohistochemistry and integrated FDG PET/MR imaging for the assessment of myocardial salvage are widely used in preclinical and clinical studies, respectively [8, 39]. However, our study was not aimed to assess cardiac scar formation/myocardial infarct size due to limited cardiac tissue samples which is a serious limitation. However, we have shown that RIPerc reduces cardiac scar formation in comparison with rats with MIR [41]. In addition, we have not investigated the signaling mechanism is causative linked to decrease in CSPG expression by RIPerc; our study is descriptive in this context. Furthermore, we can only speculate that RIPerc substantially reduces the risk of sudden cardiac death in our experimental model. Therefore, further study is warranted to clarify this issue, e.g., comparing the effect of RIPerc vs MIR on the vulnerability of isoproterenol induced arrhythmias. Of course, we cannot exclude that other factors next to CSPG play a pivotal role in RIPerc-induced early SN reinnervation following MI. Therefore, further studies are warranted to explore these potential factors and signaling mechanism.

## Conclusion

In this study, we found that remote ischemic perconditioning treatment boosted early innervation of cardiac SN following myocardial IR injury in association with the reduction in CSPG expression, whereas no net effects on the myocardial metabolism were observed.
